# A Questionnaire for the Assessment of Violent Behaviors in Young Couples: The Italian Version of Dating Violence Questionnaire (DVQ)

**DOI:** 10.1371/journal.pone.0126089

**Published:** 2015-05-19

**Authors:** Fabio Presaghi, Maura Manca, Luis Rodriguez-Franco, Giuseppe Curcio

**Affiliations:** 1 Department of Social and Developmental Psychology, Sapienza University of Rome, Rome, Italy; 2 Department of Personality, Assessment and Psychological Treatment. University of Seville, Seville, Spain; 3 Department of Life, Health and Environmental Sciences, University of L’Aquila, L’Aquila, Italy; University of Bologna, ITALY

## Abstract

In the last years, intimate partner violence (IPV) became a relevant problem for community and for social life, particularly in young people. Its correct assessment and evaluation in the population is mandatory. Our objectives were: Confirm factor structure of Dating Violence Questionnaire (DVQ) and investigate its convergent and divergent validity. The DVQ along with other personality measures were filled by a sample of 418 university students (Females = 310) of average age of 23 y.o. (SD = 4.71). A subsample of participants (223 students) consented in being involved also in retest and filled also the Revised Eysenck Personality Questionnaire (short form) and a brief scale for describing the behavior of the (past) partner after the breaking of the relationship (BRS). The 8-factor structure, with respect to the two other competing models, reported better fit indexes and showed significant correlations with other personality measures. Personality traits, both Neuroticism and Psychoticism, correlated with Sexual Violence, while Detachment correlated only with Neuroticism and Coercion, Humiliation and Physical Violence correlated with only Psychoticism. Extraversion did not report significant relationships with any of the 8 DVQ factors. Also the predictive validity of DVQ was satisfactory with the partner violent reaction to the break of relationship predicted positively predicted by Coercion (b = 0.22) and by Humiliation (b = 0.20) and negatively by Emotional Punishment (b = -0.18). The present results indicate a good factor structure of the questionnaire, and interesting correlations with personality traits, allowing to identify psychological aspects with a predisposing role for anti-social aggressive behaviors. Further studies will be aimed at ascertaining other possible determinants of intimate partner violence and the weight of cultural aspects.

## Introduction

Intimate Partner Violence (IPV) or domestic violence, consists of physical, psychological and sexual forms of abuse as well as controlling behaviors against an intimate partner. Psychological IPV refers to offensive or degrading behavior toward the partner: threats, ridicule, and withholding affection. Psychological IPV has a higher prevalence than physical IPV and has been identified as a correlate and antecedent to physical IPV [1][2]. It has recently evolved from being considered a circumscribed, domestic problem involving the private life of citizens, to a great and relevant problem for community and for social life [2].

The relevance of this issue at both psycho-social and political level brought several national and international association to investigate more in depth this issue [3]. In the more recent years an increasing amount of publications attempted to deal with this phenomenon, also as a consequence of several initiatives to create State agencies and departments dedicated to this issue [4]. Heise’s ecological framework [5] claims that the right approach to this phenomenon have to focus on its complexity and should take into consideration the different levels of IPV, i.e. individual, family/relationship, community and societal. Based on this premises, several investigations have been proposed trying to assess the specific risk factors for IPV, as for example the role of perpetrators’ alcohol consumption [6] the socioeconomic status [7] or childhood experiences of violence [8] or personality factors that predispose aggressive and anti-social behaviors [9].

IPV (specifically, dating violence) has been also reported in cross-sectional studies [10][11] as having a non trivial incidence and relevance (ranging from 61.7% for males to 64.7% for females) in teenagers and youth (as early as 13 y.o for females and 15 y.o for males) experiencing multiple types of dating violence (controlling behavior, put downs/name calling, pressured sex, insults, slapped/hits, and threats). Moreover longitudinal studies found that women victims of sexual assault in adolescence were at greater risk of revictimization in subsequent years [12] and that the most consistent predictors of violence and of its timing onset were the number of romantic partners and the early sexual debut [13]. Finally multi-country study [14] reported that even tough most of the studies on IPV during adolescence were conducted in USA their results are directly generalizable to other context. For example in industrialized countries IPV is associated with witnessing IPV during childhood, atypical family structures, multiparenting and abuse, while in non industrialized countries IPV was found to be associated with economic difficulties and early marriage [14]. Also the rates of IPV may change as function of context.

For example, the FRA study [15] reported than out of all women who have a (current or previous) partner, 22% have experienced physical and/or sexual violence by a partner since the age of 15. More of such studies are needed as are directed toward the identification of predisposing factors (personality dimensions, cultural aspects, etc) allowing for prevention of IPV [16].

The Conflict Tactics Scale (CTS) proposed by Straus in the 1979 [17] represent the first “tool to look behind closed doors” [2]. The CTS was the first questionnaire able to highlight that violence very often occurs within the context of family context. In its original form, CTS classified intimate violence in only two forms, physical and verbal/psychological [17], while its revised and improved version (CTS-2) included two new sub-scales, i.e. sexual coercion and seriousness of injuries [18]. This revised version was more complete but, as admitted by the author himself, failed to replace the original version [19]. Several other instruments followed the CTS, and today does exist a flourishing literature with several questionnaires and scales, very often well validated and with satisfactory factorial structure [20].

The most frequent classification present in the literature divides maltreatment into physical, psychological and sexual abuse [21], but in a recent critical revision it has been observed that results from factor analyses rarely match this factor structure [22].

A more recent questionnaire on dating violence, the Dating Violence Questionnaire (DVQ; formerly *Cuestionario de Violencia entre Novios*, CUVINO), has been developed in the last years by Rodriguez-Franco and coworkers [23][4][24] and validated in Spanish, Mexican and Argentinean students [24], as well as in American youths [25]. This questionnaire has been developed to assess victimization in young people and has shown a more specialized factor structure, with 8 factors for describing the abuse (Emotional Punishment; Coercion; Detachment, Physical; Derision; Humiliation; Instrumental; Sexual). This factorial structure has been replicated in both the study of 2007 (on 709 students) and on the study published in 2010 (in more than 5000 individuals coming from three different Spanish-speaking countries.

Provided the psychometric strengths and potentialities of DVQ, considered the fact that it has been developed for young people and thus its potential value for guiding prevention programs, and because of possible similarities between Italian and Spanish culture, our aim is to adapt the DVQ to Italian language. Moreover, to further inspect the convergent and divergent validity of the DVQ factors, their relationship with main personality factors, like Eysenck’ Psychoticism and Neuroticism, that are known to be linked to antisocial behavior will be investigated.

Furthermore, we intend to verify which DVQ factors are best predictors of the violent behavior consequent to the breaking of the relationship.

## Materials and Methods

### Participants

A sample of 418 participants (Males = 92; Females = 310; 16 participants did not indicate the gender) accepted to participate in the study; the mean age was of about 22 y.o. (SD = 1.88, Min-Max = 16–26). In particular our sample was comprised of late adolescents (16–21) and young adults (21–26). Due to the excessive number of missing responses, 35 participants were excluded from the analysis. So the final sample size was 383.

Of the whole sample, 223 participants (75 Males; 134 Females; for 14 participants this information was missing) accepted to participate in the retest phase (about one month later) and filled in again the questionnaires; their mean age was approximately 22 (SD = 1.45, Min-Max = 16–26). The sample was further reduced due to missing responses (84 participants returned questionnaires with an excessive number of missing responses). So the final retest sample was of 139 participants.

### Procedure

Participants were recruited among University students attending regular Psychology courses and received credits for participation in the study. All students filled in the Italian version of DVQ and a demographic information sheet both at the beginning of the course and after three months. In this second phase, together with DVQ participants also filled in construct and predictive validity scales (on personality and broken relationship).

To obtain the Italian version of the DVQ, the questionnaire was translated into Italian by two experienced researchers. The translation was then evaluated by two independent experts in the field of Social and Developmental Psychology. Finally, one Spanish mother-tongue translator back-translated the questionnaire from Italian to Spanish, to evaluate coincidence between the original and translated versions.

The study protocol was approved by the local Ethical Review Committee and conducted at the Department of Life, Health and Environmental Sciences of the University of L’Aquila, according to the principles established by the Declaration of Helsinki. Written informed consent was obtained from all participants before the study. Written informed consent was also obtained from the next of kin, caretakers, or guardians on behalf of the minors/children enrolled in the study

### Measures

The *Dating Violence Questionnaire* (DVQ)[23][4] is a 42 items questionnaire in which participants rate how often (0 = Never, 4 = Almost always) his/her partner put into practice a list of abusing behaviors that were classified in 8 different types, derived from Exploratory Factor Analysis. The eight factors were coined: Detachment (D; example items: “Has ignored your feelings”; “He/She doesn't feel responsible for what happen within the relationship nor for what happen to both of you”), Humiliation (H; example items: “Humiliate you in public”; “Insult you in front of your friends or parents”), Sexual Violence (S; example items: “You feel as if you have to have sex with him/her just to do not give him/her explanations”; “Force you to get naked when you don't want to”), Coercion (C; example items: “Tells you about your imaginary romantic relationships”; “He threatens you to suicide or to harm him/her-self if you break with him”), Physical Violence (P; example items: “Punched you”; “Harmed you with an object”), Derision (Der; example items: “Derides men or women”; “Ridicules or insults men or women”), Emotional Punishment (EP; example items: “To punish you he denies you his/her support, affect or esteem”; “He/She threats you to break with you”), Instrumental Violence (I; example items: “He/She robbed you”; “He/She indebted you”). Such a factorial solution explained 51.3% of variance, while with respect to reliability alpha values ranged between 0.58 and 0.81 [4].

The *Eysenck Personality Questionnaire-Revised Short form* (*EPQR-S*)[26] included 48 Yes-or-Not items assessing three main personality domains: Extraversion (12 items), Neuroticism (12 items), Psychoticism (12 items) while the remaining 12 items were devoted to the lie scale (Cronbach alpha for Psychoticism: 0.55; Extraversion = 0.63; Neuroticism = 0.76).

Finally, the *Breaking Relationship Scale* (*BRS*, data not published) aimed at evaluating the (past) partner negative reaction to the broken relationship, through a brief scale of 7 items asking the participants to describe the reaction of their partner when the relationship was broken based on presence/absence of some behaviors (i.e., “He/she sent me aggressive or obscene messages”, “He/she called me without speaking”, “He/she spied me”, “He/she tried to put me in bad light with my friends”, “After the break of the relationship I was in a state of alertness”, “After the break, I changed my habits”, “I changed my cell number”). Cronbach alpha was satisfactory (alpha = 0.84).

### Analysis strategy

A Confirmatory Factor Analysis (CFA) was performed for both versions of the DVQ items (rated in frequency and in degree of distress) with the primary objective to confirm the 8-factors structure proposed by Rodriguez-Franco and colleagues [23][4]. This factor structure was compared with alternative factor structures: a classic general three factor structure derived from CTS and CTS-2 questionnaires [17][18] and a factor structure considering the 8 first-order factors explained by two 2nd-order factor structures. DVQ items were considered as variable with ordered categories. For evaluating the fit of the investigated models, the significance of the Chi-square statistic, along with a series of other fit indexes and their corresponding accepted cut-off standards [27][28] were considered and in particular: the Comparative Fit Index (CFI> = .95), Tucker-Lewis Index (TLI > = .95), Root Mean Square Error of Approximation (RMSEA < = .08) and Weighted Root-Mean-Square Residual (WRMR < = .90).

Reliability, in terms of internal consistency (Cronbach alpha) and temporal stability (test-retest) have been also assessed. Spearman correlations were computed for convergent and divergent validity and due to the high number of pairwise comparisons, a Bonferroni correction will be considered to control for type 1 Error increase.

## Results

Shapiro-Wilks test for normality of distributions of each 42 DVQ items resulted all significant (and ranged from a minimum of S-W = 0.0746 of item 4, to a maximum of A-W = 0.7627 of item 25) indicating a moderate to high deviation from normality.

About 12,7% (N = 53) of participants reported that in the past they have been afraid of their partner (item 43: *Have you ever been afraid of your partner*?), and 27.7% of participants (N = 116) reported feelings of being trapped in the relationship (Item 44: *Did you ever feel the sensation of being trapped in your romantic relationship*?), while 71 participants (17.0%) reported the experience of being maltreated by their partner (Item 45: *Did you ever feel maltreated by your partner*?). Surprisingly about 54.3% (N = 227) of the participants reported to know someone that was maltreated by his/her partner (item 46: *Do you know someone (a friend of yours or a neighborhood that was maltreated by the partner during their romantic relationship*?).

Participants reported an average relationship duration of approximately 40 months (Me = 27 months, SD = 47.24, Min-Max = 1–444; N = 382) with an average age of onset of the relationship that is 20 y.o. (Me = 20; SD = 3.03; Min-Max = 12–40; N = 405). About 21.7% (N = 91) of the participants reported one or more attempts to break the romantic relationship with the partner, and 26 participants received some help from someone in order to break his/her relationships, and they reached the results in 4 month on average (Me = 3, SD = 4.47; Min-Max = 0–24).

### Confirmatory Factor Analysis

**Fig 1:**
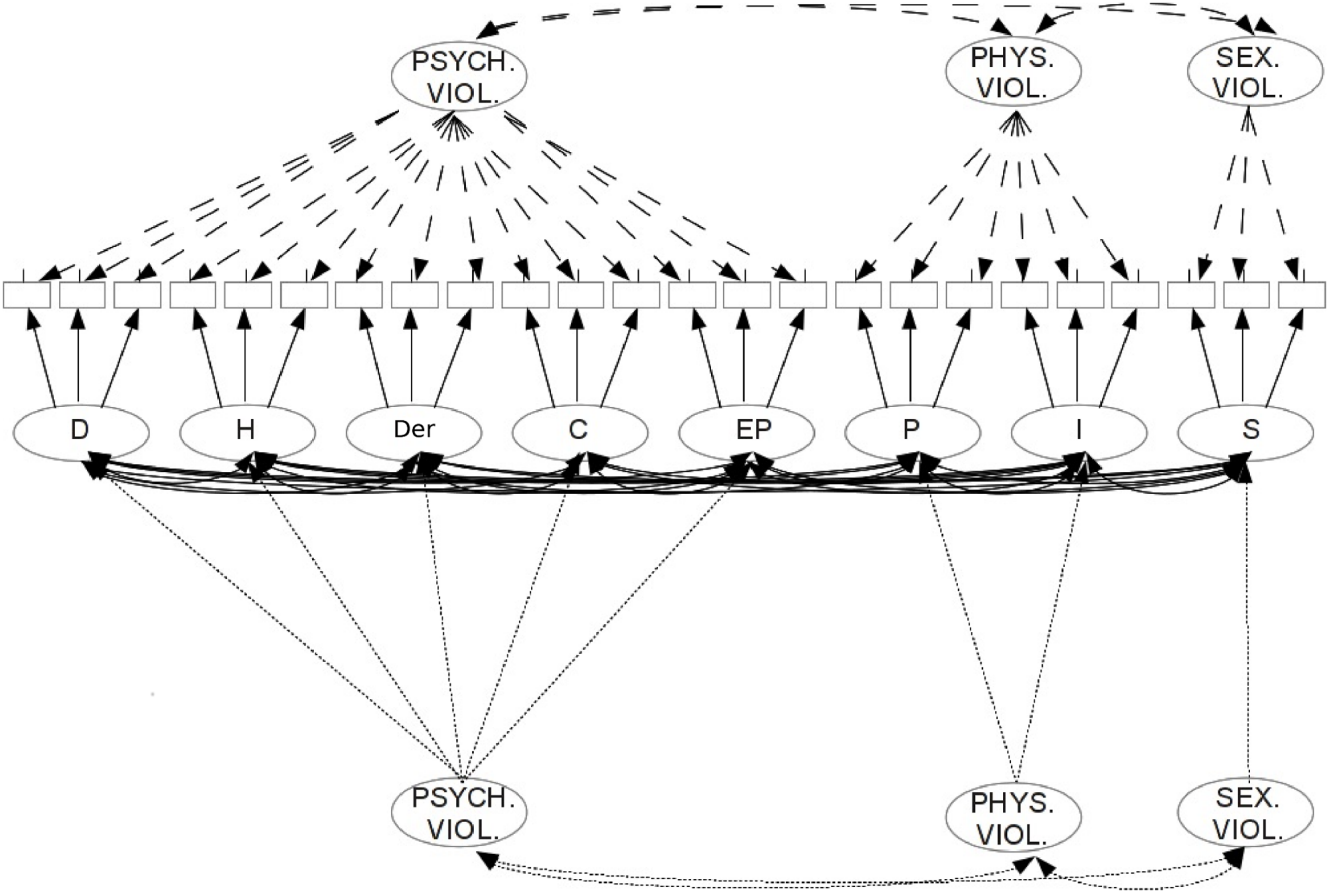
The three compared models: the original 8-factors model (a center of the figure with path shown as continuous line); the three-factor model (in the upper part of figure and shown with dotted-path lines); and the second-order factor model (shown at the bottom of figure with fine-dotted-path lines). Note: D = Detachment; H = Humiliation; S = Sexual Violence; C = Coercion; P = Physical Violence; Der = Derision; EP = Emotional Punishment; I = Indirect Violence.

A series of confirmatory factor analysis were performed (see Fig 1) investigating competing factor structure including the eight first-order factors indicated by Rodriguez-Franco and coworkers [4]. In this model all factors correlate with each other, but it can be hypothesized that these correlations maybe explained by second-order factors. So as a second model with 5-first-order factors (D, H, Der, C, EP) loading on a-second order factor (Psychological Violence) and two-first order factors (P, I) loading on Physical Violence and with the Sexual Violence reverberating the 1st order factor, was derived. This three factor structure, finally, was also tested considering that the DVQ items maybe mapped directly on these three factors: so items of D, H, Der, C, EP factors loaded directly on the Psychological Violence factors; while items describing P and I loaded on the Physical Violence factor and finally the S items loaded on this same factor (see Fig 1).

Because the DVQ items were considered as variables with ordered categories, a Robust Weighted Least Square estimator was used to estimate the model [29].

Comparing the fit statistics for all investigated models (Table 1) emerges that the 8-first-order factors model developed by the authors [4] was the one showing the best fit statistics.

**Table 1:** Comparison among the fit indexes of the three hypothesized models carried out on the whole sample (M-plus estimation method WLSMV).

	χ^2^	df	CFI	TLI	RMSEA	WRMR
8 first-order factors	1142.40	791	0.95	0.95	0.033	1.11
3 first-order factors	1251.67	816	0.94	0.94	0.036	1.22
second-order factors	1304.68	811	0.94	0.93	0.038	1.27

As shown in Table 2 all loadings are high and significantly different from zero on their respective factor.

**Table 2:** Standardized factor loadings of 1st- and 2nd-order factors at confirmatory factor analysis.

	D	H	S	C	P	Der	EP	I
dvq6a	0.525**							
dvq14a	0.651**							
dvq22a	0.628**							
dvq30a	0.775**							
dvq32a	0.572**							
dvq33a	0.803**							
dvq37a	0.911**							
cuvdvq7a		0.779**						
dvq15a		0.898**						
dvq23a		0.740**						
dvq31a		0.775**						
dvq36a		0.868**						
dvq40a		0.729**						
dvq41a		0.796**						
dvq2a			0.583**					
dvq10a			0.707**					
dvq18a			0.829**					
dvq26a			0.798**					
dvq34a			0.815**					
dvq39a			0.915**					
dvq1a				0.643**				
dvq9a				0.615**				
dvq17a				0.494**				
dvq25a				0.364**				
dvq38a				0.665**				
dvq42a				0.930**				
dvq5a					0.756**			
dvq13a					0.882**			
dvq20a					0.850**			
dvq21a					0.982**			
dvq29a					0.854**			
dvq3a						0.663**		
dvq11a						0.770**		
dvq19a						0.727**		
dvq27a						0.927**		
dvq35a						0.793**		
dvq8a							0.692**	
dvq16a							0.877**	
dvq24a							0.708**	
dvq4a								0.932**
dvq12a								0.680**
cuv28a								0.773**

** p < 0,01;

Legend: D = Detachment; H = Humiliation; S = Sexual Violence; C = Coercion; P = Physical Violence; Der = Derision; EP = Emotional Punishment; I = Instrumental Violence.

### Reliabilities and Construct Validity of the 8-factors model

Reliability of the 8-factor model was evaluated in terms of both internal consistency and temporal stability (test-retest). Cronbach's alphas at the first occasion ranged from a minimum of 0.58 (Instrumental) to a maximum of 0.84 (Humiliation) and at the second occasion ranged from a minimum of 0.52 (Emotional Punishment) to a maximum of 0.93 (Humiliation). Instead test-retest coefficients ranged from 0.31 of Derision to 0.66 of Sexual Violence.

Spearman correlation coefficients were calculated contrasting each factor with all remaining factors for both occasions (Table 3,). In general all factors reported significant and positive correlation coefficients, with the exclusion of Instrumental at the second wave that reported no-significant correlations with all factors. Detachment was mainly related to Humiliation, Coercion and Emotional Punishment at both observations. Humiliation correlated highly and positively also with Sexual Violence, Coercion, Derision and Emotional Punishment at both the test and retest. Sexual violence was further related with Coercion and Derision while Physical Violence was mainly related with both Derision and Instrumental Violence. Finally Derision reported also a high correlation with Emotional Punishment. The pattern of correlations resembles the pattern results observed in past research [4].

**Table 3:** Spearman correlations among the eight DVQ factors for the first observation (N = 383; below the main diagonal) and the second (N = 139; upon the main diagonal).

	D	H	S	C	P	Der	EP	I	Cronbach alpha
D	0.507**	0.438**	0.290**	0.600**	0.277**	0.455**	0.442**	0.211	0.82
H	0.494**	0.520**	0.202	0.453**	0.401**	0.393**	0.478**	0.110	0.93
S	0.440**	0.349**	0.665**	0.292**	0.313**	0.298**	0.280**	0.214	0.79
C	0.433**	0.418**	0.355**	0.453**	0.283**	0.429**	0.472**	0.049	0.81
P	0.208**	0.404**	0.166**	0.4260**	0.510**	0.192	0.383**	0.178	0.78
Der	0.410**	0.472**	0.365**	0.372**	0.222**	0.3311**	0.492**	0.073	0.77
EP	0.473**	0.473**	0.356**	0.415**	0.256**	0.420**	0.579**	0.111	0.52
I	0.266**	0.189**	0.195**	0.141**	0.350**	0.145	0.159**	0.418**	0.74
Cronbach alpha	0.77	0.84	0.81	0.69	0.72	0.69	0.68	0.58	--

Test-retest reliabilities are shown on the main diagonal. Cronbach alphas for the first observation (N = 383) are presented in the last row, while those for the retest are presented in the last column (N = 139).

** Bonferroni corrected-p (α = .05; number of comparisons = 28) <0.002;

Legend: D = Detachment; H = Humiliation; S = Sexual Violence; C = Coercion; P = Physical Violence; Der = Derision; EP = Emotional Punishment; I = Instrumental Violence.

Finally, Table 4 shows descriptive statistics of the 8 DVQ factors as a function of age and gender.

**Table 4:** Descriptive statistics (M and SD) of the 8 DVQ factors.

	Late adolescents (< = 21)				Young adults(>21)			
	Male (N = 27)		Female (N = 115)		Male (N = 57)		Female (N = 168)	
	M	SD	M	SD	M	SD	M	SD
D	3.07	2.88	2.69	3.41	2.63	3.34	3.05	3.70
H	2.56	2.91	1.98	3.51	2.61	3.67	1.96	3.42
S	1.15	2.30	1.13	2.67	0.91	1.29	1.07	2.20
C	3.56	3.52	2.39	2.32	2.79	2.76	2.58	3.26
P	1.30	2.28	0.37	1.14	1.19	1.99	0.30	0.95
Der	1.81	2.39	1.50	2.01	1.56	2.43	1.38	1.98
EP	1.96	2.21	0.92	1.27	1.82	1.83	1.01	1.52
I	0.41	1.08	0.10	0.48	0.26	1.48	0.07	0.37

Legend: D = Detachment; H = Humiliation; S = Sexual Violence; C = Coercion; P = Physical Violence; Der = Derision; EP = Emotional Punishment; I = Instrumental Violence.

### Convergent and divergent validity correlations

Spearman correlations between the 8 DVQ factors and the EPQ personality traits ranged from modest to moderate with Psychoticism correlating positively and modestly with Sexual Violence (rho = 0.189, p < 0.01), Coercion (rho = 0.188, p < 0.01), Physical Violence (rho = 0.173, p < 0.01) and Humiliation (rho = 0.168, p < 0.01). Neuroticism correlated positively with Sexual Violence (rho = 0.198, p < 0.01) and Detachment (rho = 0.183, p < 0.01). Extraversion did not show significant correlation with any of the 8 DVQ Factors.

### Predictive validity

A hierarchical multiple regression was performed to investigate the differential contribution of the 8 DVQ factors with respect to the 3 Eysenck Personality factors in predicting partner violent reaction to the break of relationship (BRS score). Results showed that the 3 EPQ factor alone did not explain the BRS score (R^2^ = 0.01, F(3, 168) = 0.78, p = 0.51), while by adding the 8 DVQ factors to the equation, a significant percentage of BRS scores was explained (R^2^ = 0.125, F(11, 160) = 2.08, p < 0.05). Both Coercion (b = 0.22, p < 0.05) and Humiliation (b = 0.20, p = 0.05) predicted a violent partner break of the relationship, while the Emotional Punishment showed the opposite effect (b = -0.18, p = 0.057).

## Discussion and Conclusions

As WHO declared in 2013[30], violence is not only a political and law issue but mainly a public health problem. Such a kind of violence presents a strong gendered nature, since the highest percentage of intimate violence is perpetrated towards girls and women at different levels: individual, relationship, community and societal [5][31]. Several previous investigations showed a significant gap among adult and adolescent/young population research [32][21][20].

Assessment resources in this field, in which there is a noticeable difference between those reported for adult and adolescent population [33][21][20], is needed. And an assessment tool specifically developed for the young and adolescent population in their dating relationships seems to be necessary. Romantic relationships may have an influence on many aspects of adolescent development such as family relationships, peer relationships, identity development, academic performance and the development of sexuality [34].

Our results support the factor structure reported in previous studies [4] in Spanish and Mexican samples, confirming the structure of eight factors in a complex structure that shows a pattern correlations between factors which coincides with the very complex dynamics of IPV, as reflected in both the second-order factors as the results of additional questions such as “Have you ever been afraid of your partner?” (item 43), “Did you ever feel the sensation of being trapped in your romantic relationship?” (Item 44), and “Did you ever feel maltreated by your partner?” (Item 45) that have been analyzed in other studies [24][35].

The DVQ has shown to maintain the original factor structure despite the social and cultural differences between different countries (Spain, Mexico, USA, Italy and others). This requires a cross-cultural study to determine the relationship between other variables and dating violence. In our study, tentatively, we analyzed the relationships between personality traits and violence with promising results. The questionnaire factors also showed interesting correlations with personality traits known to play a predisposing role to anti-social aggressive-based behaviors [9]. General peer violence and delinquent behaviors are strongly associated with aggression in dating relationships, demonstrating how peer environments foster and support emerging dating dyads [36]. In particular a more coercive and instrumental violence seems to be more related to a psychoticism personality trait, while a more detached and physical violence seem to be more typical of neurotic trait. Physical aggression in teen romantic relationships may be associated with the learning of maladaptive conflict resolution techniques that will be used in future romantic relationships. This indicates the complex dynamics of IPV, but should be considered other determinants such as gender role and others. Physical aggression on adult partner violence is preceded by verbally or emotionally abusive behavior earlier in the relationship; thus, adolescents who become aggressive in their dating relationships may set a course for a continuing pattern of hostility and aggression toward others. It would be unlikely that a small number of variables allowed predicting partner violence and further research would reveal possible hidden drives. Use of aggression against a dating partner is predictive of an individual's use of aggression against a subsequent dating partner, was supported for males but not for females. These findings suggest that males and females may be aggressive against intimate partners for different reasons. The main limitation of the instrument is related to the fact that DVQ is aimed to measure intimate violence among people that is not married or that is not constrained by the fact that they live together. On the other hand, this aspect could allow to disentangle some interesting effects related to violence in youngest couples. Another limitation of the instrument is related to the restriction to young people who have had partners for at least one month, with the intrinsic exclusion of some other types of violence, mainly occurring in occasional relationships.

Our results show the relevance of Dating Violence Questionnaire in an Italian sample including late adolescents and young adults. With respect to the 31.9% of women (aged 16–70 ys) that received violence at least one time in their life [37], here we observed that 17% of the participants sample reported the experience of being maltreated by their relationship partner in a longer time duration (average of approximately 40 months) with an average age of onset of the relationship that is 20 y.o. and 21.7% (N = 91) of the participants reported one or more attempts to break the romantic relationship with the partner. Aggression in adolescent romantic relationships is an important problem because of its prevalence because affecting 25% to 50% of adolescents [38]. If we consider that the relationships at this age can be considered as roles, behaviors and attitudes learning that foster violence in adults, the relevance of investigating violence in this life period can be understood and highlighting the relevance to develop primary prevention programs for these individuals. Attitudes that support aggression as a justifiable solution to conflict among couples have often been linked to reports of dating aggression [39]. Characteristics of previous relationships may be influential in continuance of aggressive response to conflict within a new dating relationship.

A larger number of studies is needed, particularly in Europe, where this field of research is only limitedly explored. In this way it could be overcome also some of the limitations of the present study. It would be interesting, for example, explore the levels of awareness about the abuse of young women, or the relationship between the levels of tolerance for abuse and frequency of violent acts committed in the couple. Such studies would allow designing prevention programs for adolescents and youth, helping to prevent violent behavior patterns were consolidated during adulthood. Moreover, since the elevated mean age of the present sample, further studies should involve younger samples, namely early and late adolescents. Finally, another interesting aspect that is not addressed by this paper is the possibility to investigate in the two genders different way to use violence, doing this in both hetero- and homosexual couples. Information on the prevalence of consequences of IPV for non-heterosexual victims, in fact, could be crucial during the development of such shelters, especially if non-heterosexuals experience a higher likelihood of negative consequences [40].

Our results also point to the need for revision of some factors, especially in regard to Instrumental factor. The assessment tools are dynamic techniques that require continual checks. The team that originally developed the questionnaire right now is improving the wording of the items on some factors, especially the instrumental violence and developing a new factor related to violence through media online. All these changes will surely improve the “ecological” usefulness of this instrument and its potentiality as a preditor for future anti-social aggressive behaviors in the intimate relationships.
